# Effect of Isopropanolic *Cimicifuga racemosa* Extract on Uterine Fibroids in Comparison with Tibolone among Patients of a Recent Randomized, Double Blind, Parallel-Controlled Study in Chinese Women with Menopausal Symptoms

**DOI:** 10.1155/2014/717686

**Published:** 2014-03-02

**Authors:** Sisi Xi, Eckehard Liske, Shuyu Wang, Jianli Liu, Zhonglan Zhang, Li Geng, Lina Hu, Chunfeng Jiao, Shurong Zheng, Hans-Heinrich Henneicke-von Zepelin, Wenpei Bai

**Affiliations:** ^1^Department of Gynecology, The First Hospital of Peking University, Beijing, China; ^2^Departments of Life Sciences, Technical University of Braunschweig, 38106 Braunschweig, Germany; ^3^Department of Gynecology, Jiangsu Province People's Hospital, Nanjing, China; ^4^Department of Gynecology, The General Hospital of PLA, Beijing, China; ^5^Department of Gynecology, The Third Hospital of Peking University, Beijing, China; ^6^Department of Gynecology, West China Second Hospital of Sichuan University, Chengdu, China; ^7^Biometrical Department, Excel Pharma Studies, Beijing, China; ^8^Schaper & Brümmer GmbH & Co. KG, Preclinical and Clinical Research, 38259 Salzgitter, Germany

## Abstract

*Objective.* Effect of isopropanolic *Cimicifuga racemosa* extract (iCR) on uterine fibroid size compared with tibolone. *Method.* The randomized, double-blind, controlled study in China enrolled 244 patients aged 40–60 years with menopausal symptoms (Kupperman Menopause Index ≥ 15). The participants were treated with either iCR of 40 mg crude drug/day (*N* = 122) or tibolone 2.5 mg/day (*N* = 122) orally for 3 months in 2004. Now, we investigated the subset of all women (*N* = 62) with at least one uterine fibroid at onset of treatment for the effect of iCR (*N* = 34) on fibroid size compared with tibolone (*N* = 28) by transvaginal ultrasonography. *Results.* The median myoma volume decreased upon iCR by as much as −30% (*P* = 0.016) but increased upon tibolone by +4.7%. The percentage of volume change, mean diameter change and geometric mean diameter change of the iCR group compared to tibolone were statistically significant (*P* = 0.016, 0.021, 0.016 respectively). *Conclusion.* Our results suggest that iCR (Remifemin) is a valid herbal medicinal product in patients with uterine myomas as it provides adequate relief from menopausal symptoms and inhibits growth of the myomas in contrast to tibolone.

## 1. Introduction

Most women in menopausal transition have symptoms such as hot flashes, night sweat, and associated sleep impairments. Menopausal symptoms are closely related to estrogen deficiency so that estrogen is an effective way to relieve menopausal symptoms. However, the treatment of menopausal symptoms in women with uterine fibroids is still debated controversially since previous studies suggested that estrogens play a role in the growth of uterine fibroids [1]. Uterine fibroids are the most common benign tumors in females which could be found in 77% of hysterectomy specimens [2] and there is some hesitation to use hormones in these patients. Thus, there is a demand for safe and effective treatments of menopausal complaints for the large numbers of women with myomas.

Tibolone and iCR are commonly used to relieve menopausal symptoms. As a hormone-like medicine, tibolone binds to estrogen, progesterone, and androgen receptors. It acts agonistic and/or antagonistic on hormone receptors, and is thus described as Selective Estrogen Receptor Modulator (SERM [3]). Tibolone may stimulate the uterine smooth muscle and endometrial, but it is inconclusive if this drug could increase the risk of uterine fibroids or endometrial cancer [4]. Remifemin contains a standardized isopropanolic extract of the rootstock of *Actaea* (i.e., *Cimicifuga*) *racemosa* (iCR). Due to its positive benefit-risk ratio, this herbal medicine became a new choice for the treatment of menopausal symptoms. Safety data on iCR are available from more than 11,000 patients investigated in clinical studies for up to 12 months treatment (sum of 21 clinical studies, all published). However, more studies about the safety of iCR are said to be desirable [5]. Particularly, data on effects of iCR on myomas have been lacking. The aim of our investigation was to evaluate in this subgroup analysis the effects of tibolone and iCR treatment (12 weeks) on uterine fibroids in women treated for their menopausal complaints.

## 2. Material and Methods

Our research group had published a paper “Efficacy and tolerability of a medicinal product containing an isopropanolic black cohosh extract in Chinese women with menopausal symptoms: A randomized, double blind, parallel-controlled study versus tibolone” in Maturitas (2007) which had been conducted at five hospitals in China [6]. Among those previous study patients, 34 women in the iCR group and 28 women in the tibolone group had uterine fibroids at onset of therapy. These 62 women were included to the current investigation, which analyzed the change of the leiomyoma sizes before and after the 3-month treatment with tibolone or iCR being indicative for the safety of these medications even in the presence of uterine fibroids. The original study had obtained the approval of the Ethics Committee and each participant had signed informed consent. The study had been registered in http://www.clinicaltrials.gov/ (identifier: NCT00299364) and approved by the Chinese State Food and Drug Administration. For entrance criteria and other details on methods and setting, please see our previous paper [6]. In our research, 244 subjects who met the requirements were enrolled and randomized, 122 per treatment group. Two hundred and eighteen subjects (89.3%) completed this trial.

At the first visit, women underwent:

- general and gynaecological anamnesis,
- physical examination,
- ultrasound evaluation on the radial line of the uterine for measuring the size of myoma if existing and the endometrial thickness,
- ultrasound of the breast,
- cervical smear,
- clinical interview for menopausal symptoms.

In the same day, patients provided blood samples for the determination of follicle stimulating hormone (FSH), estrogen (E_2_), standard hematology, and biochemistry. Urine samples served for urinalysis. Our previous paper reported the primary results on efficacy in menopausal complaints and safety in detail.

The patients were treated for twelve weeks with iCR (Remifemin, bulk-batch number 422450; manufactured by Schaper & Bruemmer, Salzgitter-Ringelheim, Germany) or tibolone (produced by Zizhu Pharm, Beijing, China, bulk-batch number 20040416). Scheduled study visits were as follows: visit 1 at study entry, visit 2 after 4 weeks, and visit 3 after 12 weeks of treatment. On each follow-up visit clinical variables such as KMI, vital signs, body weight, concomitant diseases, adverse events, and concomitant medication were documented in the CRF. Blood sampling for standard hematology and biochemistry and a black and white ultrasound evaluation of endometrial thickness were conducted before onset and at the end of treatment.

Among the 218 subjects who completed the trial (iCR group 110 subjects/tibolone group 108 subjects), there were 34 women in the herbal group with uterine fibroids and 28 women in the tibolone group with uterine fibroids at onset of treatment. In the investigations for this paper, we focused on the information about the fibroids of these 62 women and made statistical analysis to detect any effect of the two treatments on the uterine fibroid size of the women suffering from menopausal complaints.

For the ultrasound measurements, doctors in the five centers were specially trained before the study. The diameters of the ellipsoid sphere (myoma) in three rectangle dimensions, *d*_1_, *d*_2_, and *d*_3_, (craniocaudal length, transverse width, and anterior/posterior diameter) were measured. Only the individually largest fibroid for each woman was included in our analysis in order to ensure consistency of measurement during followup. We calculated the mean diameter as *d*_mean_ = (*d*_1_ + *d*_2_ + *d*_3_)/3, the geometric mean diameter as $d_{\text{geomean}}=\sqrt[{3}]{\left(d_{1}\times d_{2}\times d_{3}\right)}$, and the individually largest fibroid volume using the formula for an ellipsoid sphere (*V* = (*π*×*d*_1_ × *d*_2_ × *d*_3_)/6). Percent of mean/geometric mean diameter change was calculated as ((*d*_*t*3_ − *d*_*t*1_)/*d*_*t*1_) × 100% (where *d*_*t*1_ and *d*_*t*3_ are the mean/geometric mean diameter measured at visit 1 and visit 3). Percent of volume change per 12 weeks was calculated as ((*V*_*t*3_ − *V*_*t*1_)/  *V*_*t*1_) × 100% (where *V*_*t*1_ and *V*_*t*3_ are volume measured at visit 1 and visit 3) which can be transposed to yearly volume change by multiplication with 4.

Statistical software SPSS 12.0.3 was used for data analyses.

Independent-sample *t*-test was used for demographic and other baseline characteristics after normal distribution was confirmed by Kolmogorov-Smirnov test. Normal distribution was rejected for fibroid volume, difference of volume during 12 weeks, difference of diameter (both mean diameter and geometric diameter) during 12 weeks, and changes of fibroid volume (%). Due to this, nonparametric tests were used, that is, Wilcoxon signed ranks test for within group comparisons of volumes, mean diameters, and geometric diameters and the Mann-Whitney rank-sum test for intergroup comparisons at visits 1 and 3 as well as for the change from visit 1 to 3. Cross-tabulations including Fisher's exact test and logistic regression analysis were used for investigating the decrease rates of myoma size. Statistical significance for any treatment effect was tested at *α* < 0.05. Logistic regression considered putative confounders in a stepwise backward elimination procedure with the treatment group fixed in the model and nonrelevant confounders stepwise eliminated if exceeding the threshold *α* < 0.10. The starting model included body mass index (BMI), number of pregnancies, age at menarche, serum estradiol at baseline, FSH at baseline, age, duration of amenorrhea, duration of climacteric complaints, and the Kupperman Menopause Index (KMI) at baseline as putative confounders. With this procedure which keeps the independent variable “group” always in the model, the overall type 1 error (*α* < 0.05) remains preserved.

Descriptive data is presented as median and interquartiles. For normally distributed variables, additionally mean ± standard deviation (SD) is shown.

## 3. Results

The patients were from five hospitals in China (The First Hospital of Peking University (*N* = 12), the General Hospital of PLA (*N* = 13), the Third Hospital of Peking University (*N* = 13), West China Second Hospital of Sichuan University (*N* = 7), and Jiangsu Province People's Hospital (*N* = 17)). These 62 subjects all completed the study and tolerated the treatment well.

The demographic and other baseline characteristics of the subjects are listed in Table 1. At the beginning of the study (baseline), there were no significant differences between the iCR and the tibolone groups with respect to age, amenorrhea duration, BMI, number of pregnancies, age at menarche, thickness of the uterine intima, KMI, levels of serum FSH, and serum estradiol (E_2_).

**Figure 1:**
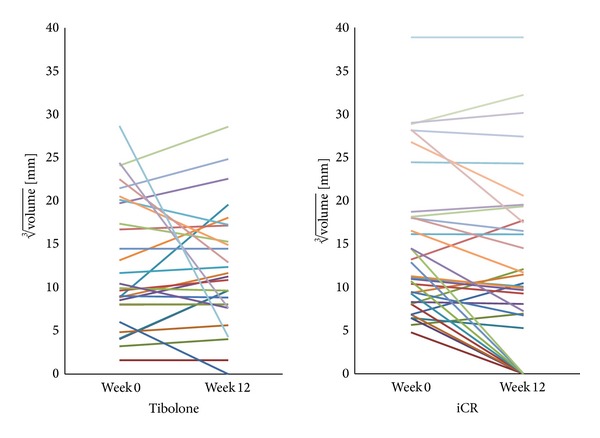
Individual data on the size of the largest myoma in each of the 34 patients in the iCR group and the 28 patients in the tibolone group.

Median volume, mean diameter, and geometric mean diameter of the myomas are shown in Table 2. In the iCR group (Figure 1), the median volume of the individually largest fibroid decreased from 1787 (IQR, 599–6107) mm^3^ at visit 1 to 1086 (IQR, 0–5991.0) mm^3^ at visit 3 (*P* = 0.085). The mean diameter and the geometric mean diameter of the individually largest fibroid per patient significantly decreased during the treatment (*P* = 0.006 and *P* = 0.006). A decrease of the myoma volume was observed in 24 women of the iCR group (70.1%). The myomas' volume changed on average by −30.3% (decrease) during the 12 weeks of treatment.

In the tibolone group (Figure 1), the median volume of the individually largest fibroid changed from 1063 (IQR, 520–8086) mm^3^ at visit 1 to 1096 (IQR, 448–4695) mm^3^ at visit 3 (*P* = 0.657). No statistically significant difference from baseline was also found for the mean diameter (*P* = 0.819) and the geometric mean diameter (*P* = 0.778) of the individually largest fibroid in the patients treated with tibolone for 12 weeks. A decrease of the myoma volume was observed in 10 women of the tibolone group (35.7%). The myomas' volume changed on average by +4.7% (increase) at 12 weeks.

The key result of this investigation is the comparison between the treatment groups regarding the changes of volume, mean diameter, and geometric mean diameter of the individually largest myoma per patient during the treatment (Table 2). The percentage of volume change in the iCR group (−30.3% decrease) was significantly superior to the one in the tibolone group (+4.7% increase) (*P* = 0.016). Also, the percentage of mean diameter change and geometric mean diameter change in the herbal group was significantly superior to the one in the tibolone group (mean diameter change *P* = 0.021, geometric mean diameter change *P* = 0.016). Moreover, the response rate (response = decrease of myoma size) in the iCR group (70.1%) was superior to the one in the tibolone group (35.7%) (*P* = 0.010).

For further exploration, the response rates were analyzed for confounding parameters. The final logistic regression model (Table 3) included an influence of the serum estradiol level at baseline and the Kupperman index at baseline and revealed the odd's ratio 0.213 (= 1/4.7) for a response in favor of the iCR group as compared to the tibolone group (*P* = 0.008). In other words, the odds of response in the iCR group was 4.7-fold the odds in the tibolone group.

## 4. Discussion

Our recent randomized, double blind, parallel-controlled trial showed that the efficacy of iCR was as good as tibolone for the treatment of menopausal symptoms, while the herbal group was superior regarding the safety profile [6]. The duration of iCR and tibolone treatment for menopausal symptoms was 12 weeks. The menopausal symptoms of the patients were significantly improved in both groups. Our current new data analysis of that study shows a significant difference between the effect of iCR and tibolone therapy on the course of the uterine fibroid size in the subset of women with uterine fibroids. During the 12 weeks treatment iCR induced a decrease of fibroid volume to a certain extent while tibolone did not.

The phytomedicine Remifemin contains the isopropanolic extract of *Actaea* (i.e., *Cimicifuga*) *racemosa *(iCR). Differentiated evaluation of extract-specific evidence on *Cimicifuga racemosa*'s efficacy and safety for climacteric complaints revealed that a positive benefit-risk profile is limited to *Cimicifuga racemosa* products holding a marketing authorization for treating climacteric complaints [7, 8]. This herb was used traditionally and for some decades iCR has been available as herbal medicinal product for alleviating menopausal symptoms such as hot flushes, night sweats, and associated sleep impairments [5]. iCR is not a so-called phytoestrogen, it does not have direct estrogenic effects [9], and it does not interfere with gynecological hormones [7] and LH pulse frequency in vivo [10]. Recent studies showed the reason why iCR could relieve neuroendocrine system symptoms such as hot flushes and others may be due to the effect on serotonin pathways [11] and the endogenous opioid system [12]. In view of such advantages, iCR attracted wide attention of patients with menopausal symptoms regardless of their menopausal status and also included patients with estrogen-sensitive tumors.

Our results show a decrease trend of the myoma volume in most women (70.1%) treated with iCR and the myoma volumes were changed on average by −30.3% (decrease) during 12 weeks of treatment. The growth of uterine fibroids is closely related to estrogen and progesterone, but there was no (or little) research published referring to effect of black cohosh in general on uterine fibroids. Uterine fibroids, endometrium, and breast are estrogen sensitive tissues in females. Fluctuations of endogenous estrogen may cause hyperplasia or atrophy of the three tissues. Previous studies investigated black cohosh for any effects on breast and endometrium. Geller et al. [13] showed that 12 months treatment with extract of black cohosh rhizome (128 mg/d standardized to 7.27 mg triterpene glycosides) did not increase the risk of the malignant change of the breast and the endometrium. Li et al. showed that iCR did not increase the risk of tumor recurrence in patients with early endometrial cancer upon 24 months treatment of their menopausal symptoms after operation [14]. In another study, Hirschberg et al. [15] showed that 6 months treatment with 40 mg black cohosh daily did not cause change of mammographic density and the breast cell proliferation. This was even further confirmed by recent meta-analyses of clinical study data on this safety aspect [16]. Another study [17] found that isopropanolic *Cimicifuga racemosa* extract does not increase the risk of breast cancer recurrence and it may be associated with prolonged disease-free survival. Our present clinical study data analysis is the first investigation on the safety of iCR on uterine fibroids. Short term treatment (3 months) is safe for women with fibroids and the volume of fibroids may even decrease during treatment, suggesting that iCR is a low risk choice for women with menopausal complaints with uterine fibroids.

Tibolone alleviates menopausal vasomotor symptoms and can help women to improve their mood. Tibolone also plays roles on relief of the discomfort of the urinary tract and the vagina, improvement of the quality of sexual wellbeing, and reduction of menopausal bone loss [18]. In our current investigation, we found an increase trend of the myoma volume in half of the women (53.6%) treated with tibolone, and the myoma volumes changed on average by 4.7% (increase) during 12 weeks of treatment. But the mean diameter and geometric mean diameter of the individually largest fibroid per patient did not show significant changes during the treatment with tibolone for 12 weeks. Uterine fibroids are sex hormone-dependent tumors and contain estrogen binding receptors and progesterone binding receptors. Recently, some evidence pointed to progesterone as major promoter of uterine fibroids development and growth [19]. Progesterone may promote the growth of fibroids as it plays roles in human body through progesterone receptors (PR) and PR can interact with growth factor signaling systems to promote proliferation and survival of uterine fibroids [19].

As tibolone has androgen effects as well as some estrogen and progesterone activity, many studies focused on the effect of tibolone on estrogen and progesterone sensitive tissues such as uterine fibroids [18–20]. The effects of tibolone on uterine fibroids in menopausal women have been evaluated in several other clinical trials. Previous studies showed that postmenopausal women taking tibolone 2.5 mg per day orally for 6 months [20, 21], 12 months [22], and 3 years [21] did not cause significant increase of fibroids but the uterine hemodynamic parameters changed as the uterine arteries' pulsatility index increased in women with fibroids [23]. There are some differences between the results of this study and previous researches. Our study involved perimenopausal women and postmenopausal women and the average estrogen level in the screening period was about 64 pg/mL (but always less than 30 pg/mL if amenorrheic interval had been less than 12 months) while the above mentioned studies on tibolone only involved postmenopausal women and excluded patients with a serum estrogen level higher than 30 pg/mL.

A natural decline in size of uterine fibroids over time is to be expected in menopausal women [19]. In a recent study on this topic, Mavrelos et al. [24] reported that the natural growth rate of the median volume of myomas was 35% per year according to their findings in 178 women aged between 25–45 years. Peddada et al. [25] found that the median growth rate of myomas was 9% per six months (equalling 19% per year). These two studies agree with our results in myoma patients in the tibolone group (increase of the volume by 4.7% per 12 weeks which is equal to 21% per year). We conclude that short term treatment (12 weeks) of tibolone does not interfere with the natural course of uterine fibroids. As tibolone has a neutral effect in growth of myomas, women taking tibolone to treat menopausal symptoms should regularly be reexamined by B-monitoring (comprising the diameter, the blood flow, the ultrasound echo intensity, the borderline between uterus myoma and uterus muscle, etc.) to check the fibroids.

The aim of the present investigation was to evaluate the effects of tibolone and iCR treatment (12 weeks) on uterine fibroids in women with menopausal complaints, and we detected significant difference between the influence of iCR and tibolone on the change of the fibroids' volume. Both drugs can help menopausal women to relieve their menopausal symptoms effectively and similarly, but iCR seems to be the better choice in alleviating menopausal symptoms in women with uterine fibroid. iCR seems to be a valid treatment in patients with uterine fibroids as it provides adequate relief from menopausal symptoms and avoids increase in uterine fibroid size, which is usually a cause of concern for the patient.

There are some limitations of this study. As we only involved 62 fibroid patients and we just evaluated the short term treatment of iCR on fibroids, the conclusion cannot yet be made that the herbal treatment can shrink the fibroids volume definitely. The evaluation of long time effect of iCR and/or tibolone in more patients could be done in a future study.

## 5. Conclusion

12 weeks treatment with iCR shows relatively low risk for women with fibroids and the volume of fibroids may decrease during treatment. Short term treatment of tibolone does not affect the natural growth of fibroids. Compared with tibolone, iCR seems to be the better choice in alleviating menopausal symptoms in women with uterine fibroid.

## Figures and Tables

**Table 1:** Demographic and other baseline characteristics (mean ± SD).

	iCR (*N* = 34)	Tibolone (N = 28)	P
Age (years)	52.6 ± 3.0 (range 47 to 60)	51.5 ± 4.6 (range 41 to 60)	0.304
Duration of amenorrhea (months)	31.5 ± 23.9	32.4 ± 25.7	0.896
BMI (kg/m^2^)	23.1 ± 2.5	23.5 ± 2.5	0.526
KMI	24.9 ± 6.1	26.2 ± 5.9	0.409
Age at menarche (years)	14.9 ± 2.1	14.3 ± 1.8	0.238
Number of pregnancies	2.6 ± 1.1	2.5 ± 1.2	0.754
Serum estradiol (mg/dL)	54.0 ± 45.6	56.3 ± 38.6	0.835
Serum FSH (U/mL)	83.7 ± 33.2	78.5 ± 29.2	0.518
Thickness of uterine intima (mm)	2.9 ± 1.2	3.1 ± 1.1	0.535

**Table 2:** Changes of volume, mean, and geometric mean diameter of the largest myoma per patient during the treatment. Median and interquartile range (IQR) are shown for the volume parameters for which normal distribution was rejected, and mean ± SD is shown for the diameter parameters for which normal distribution was not rejected. *P* values for intergroup comparison by Mann-Whitney *U* test.

Group	iCR	Tibolone	P
*N*	34	28	
Volume (mm³)			
Visit 1	1787 (559; 6107)	1063 (520; 8060)	0.35
Visit 3	1086 (0; 5991)	1096 (448; 4695)	0.91
Volume change (%)	**−30.3 (−100.0; +12.7)**	**+4.7 (−35.9; +88.3)**	**0.016**
Mean diameter (mm)			
Visit 1	18.7 ± 10.6	16.5 ± 10.7	0.37
Visit 3	15.1 ± 12.8	14.8 ± 8.5	0.85
Mean diameter change (%)	−25.6 ± 47.6	+6.6 ± 54.7	**0.021**
Geometric mean diameter (mm)			
Visit 1	18.6 ± 10.5	16.0 ± 9.2	0.35
Visit 3	14.8 ± 12.9	14.4 ± 8.9	0.91
Geometric mean diameter Change (%)	−26.6 ± 48.8	+6.1 ± 58.0	**0.016**

**Table 3:** Odds ratio of response from treatment. Response is defined as decrease of myoma size throughout the treatment. The table shows the final logistic regression model after stepwise backward elimination procedure with the treatment group fixed in the model and nonrelevant confounders stepwise eliminated if exceeding the threshold  *α* < 0.10. The starting model included body mass index (BMI), number of pregnancies, age at menarche, serum estradiol at baseline, FSH at baseline, age, duration of amenorrhea, duration of climacteric complaints, and the KMI at baseline as putative confounders.

Variable	*B*	S.E.	*P* value	Exp(*B*)
Treatment group	−1.544	0.578	**0.008**	**0.213**
Serum estradiol at baseline	0.013	0.008	0.092	1.013
Kupperman index at baseline	−0.091	0.055	0.098	0.913
Constant	4.063	1.628	0.013	58.165
